# Peri-implant and systemic effects of high-/low-affinity bisphosphonate-hydroxyapatite composite coatings in a rabbit model with peri-implant high bone turnover

**DOI:** 10.1186/1471-2474-13-97

**Published:** 2012-06-11

**Authors:** Shun Niu, Xiaorui Cao, Yan Zhang, Qingsheng Zhu, Jinyu Zhu, Ping Zhen

**Affiliations:** 1 Department of Orthopaedics, Xi Jing Hospital, The Fourth Military Medical University, Xi’an, Shaanxi, 710032, China; 2 Department of Digestive Diseases, Xi Jing Hospital, The Fourth Military Medical University, Xi’an, Shaanxi, 710032, China; 3 Department of Orthopaedics, General Hospital of Lanzhou Military Command, Lanzhou, Gansu, 730050, China

## Abstract

**Background:**

Hydroxyapatite (HA) coatings composed with bisphosphonates (BPs) which have high mineral-binding affinities have been confirmed to successfully enhance implant stability. However, few previous studies focused on HA coatings composed with low-affinity BPs or on systemic effects of locally released BPs.

**Methods:**

In this long-term study, we developed two kinds of BP-HA composite coatings using either high-affinity BP (alendronate, ALN) or low-affinity BP (risedronate, RIS). Thirty-six rabbits were divided into three groups according to different coating applications (group I: HA, group II: ALN-HA, and group III: RIS-HA). Implants were inserted into the proximal region of the medullary cavity of the left tibiay. At insertion, 2 × 10^8^ wear particles were injected around implants to induce a peri-implant high bone turnover environment. Both local (left tibias) and systemic (right tibias and lumbar vertebrae) inhibitory effect on bone resorption were compared, including bone-implant integration, bone architecture, bone mineral density (BMD), implant stability, and serum levels of bone turnover markers.

**Results:**

The results indicated that ALN-HA composite coating, which could induce higher bone-implant contact (BIC) ratio, bone mass augmentation, BMD, and implant stability in the peri-implant region, was more potent on peri-implant bone, while RIS-HA composite coating, which had significant systemic effect, was more potent on non-peri-implant bone, especially lumbar vertebrae.

**Conclusions:**

It is instructive and meaningful to further clinical studies that we could choose different BP-HA composite coatings according to the patient’s condition.

## Background

As the most effective surgical procedure for treating severe arthritis and other joint-related diseases, the number of total joint arthroplasty (TJA) increases steadily every year. Thus wear debris-induced aseptic loosening and subsequent revision surgery may be unavoidable, especially for young patients.

Considerable studies have focused on preventing aseptic loosening and enhancing implant stability in revision surgeries. BPs are a promising class of widely used drugs for implant stability due to their inhibitory effects on osteoclasts [1-6].

BPs have a P-C-P structure and two side-chains (R1 and R2). Their mineral-binding affinities are mainly influenced by R1 side-chain. In addition, R2 side-chain, three-dimensional (3D) conformation, and the orientation of the nitrogen also play a role [3]. Nancollas *et al.*[7] established a rank order of their mineral- binding affinities: zoledronate (ZOL) > ALN > ibandronate > RIS > etidronate > clodronate. The anti-resorptive potency is mainly influenced by R2 side-chain, and the rank order is: ZOL > RIS > ibandronate > ALN > pamidronate, which closely matches the order of potency on inhibiting farnesyl pyrophosphate synthase (FPPS) [3,8-10].

Due to the different affinities and anti-resorptive potencies, systemically administrated high-affinity BPs (ALN and ZOL) are effective on vertebral fractures, while low-affinity BPs (RIS) are effective on all fracture types, especially non-vertebral fractures [3,11]. The positive effects of BPs on bone-implant integration have been widely studied by being administrated either systemically (oral, intravenous or subcutaneous) [12-15] or locally (peri-implant injection or composite coating delivery) [1,2,4,5,16-22].

Peri-prosthetic high bone turnover is the major pathological response to aseptic loosening, so local BPs treatment may be preferable for local high bone turnover-related diseases. Digestive ulcer [23] and osteonecrosis of the jaw [24] are the major side effects caused by BPs treatment. High oral or intravenous dose is considered to be the major cause of these side effects. Because of the mineral-binding selectivity [25] and poor oral bioavailability [8] of BPs, most BPs will be consumed by non-peri-implant bone which has the highest turnover rate [25], and be excreted via urine [8]. Thus, patients have to take more BPs than their bone really need if they take BPs systemically. Oppositely, if BPs are administrated locally, the unnecessary consumption can be avoided, and we do not need to worry about their poor oral bioavailability. Therefore, lower drug dose than systemic treatment will be enough, and dose-depended side effects may be avoided. However, some problems should be avoided if BPs are administrated locally that local high drug concentration and massive bone compaction may impair bone-implant integration [26,27].

Recently, some studies focused on the BP-HA composite coatings to prevent aseptic loosening. Suratwala *et al.*[4] developed a ZOL-HA composite coating, which enhanced the peri-implant bone quality and implant stability in rats with aseptic loosening. However, as we know, revision patients are averagely older than the primary patients that they have higher incidence rate of systemic osteopenia or osteoporosis. Therefore, in addition to enhancing implant stability, it is better for the composite coatings to have systemic effects of bone mass augmentation and BMD increment which may benefit the recovery for revision patients. However, none of previous studies investigated or compared the systemic effects of locally released high-/low-affinity BPs.

In this long-term study, we developed two kinds of BP-HA composite coatings using either high-affinity BP (ALN) or low-affinity BP (RIS) in rabbits with peri-implant high bone turnover rate. Both local and systemic inhibitory effects on bone resorption were compared. We hypothesised that the ALN-HA composite coating is more effective on peri-implant bone while RIS-HA composite coating has more obvious systemic effects and is more effective on non-peri-implant bone.

## Methods

### Experimental design

Thirty-six male New Zealand white rabbits (3.00 ± 0.20 kg) were divided into three groups. The implant was inserted into the proximal region of the medullary cavity of the left tibia, and particles were injected around the implant to induce a peri-implant high bone turnover environment. In group I (n = 12), HA-coated implants were not composed with BPs. In group II (n = 12), HA-coated implants were composed with ALN. In group III (n = 12), HA-coated implants were composed with RIS. The study lasted for 24 weeks, and every 12 weeks six animals from each group were killed.

### Particles and implants

Commercial pure ultra-high molecular weight polyethylene (UHMWPE) particles (average 1.74 ± 1.43 μm, range 0.05-11.06 μm) were obtained from the manufacturer (Ceridust VP 3610, Clariant, Germany). Detailed parameters have been described by von Knoch *et al.*[28]. Before injection, the particles were tested using a quantitative limulus amebocyte lysate assay to assure that the endotoxin level was lower than 0.25 EU/mL.

Titanium alloy rods (Ti6Al4V, 2.5-mm diameter and 45-mm length) were plasma spray coated with HA (coating thickness = 30 μm, Ca/P = 1.67, Biomaterial Centre of Sichuan University, Sichuan, China).

To prepare the BP-HA composite coatings, 100-μg of ALN or 50-μg of RIS (potency is equivalent to 100-μg of ALN) was dissolved in 100-μL of distilled water at 60 °C. The solution was evenly dropped onto the surface of an HA coating using a micropipette, and the coating was entirely covered by the solution. Because 100-μL of solution could not be absorbed by the HA coating at once (see Additional file 1), we rotated the implant (90 degrees) every 5 minutes until there was no dripping on the implant, and then the implant was dried at 50 °C for 24 h and sterilised with gamma irradiation. Therefore, the homogenous distribution of the BP solution on the implant surface could be guaranteed.

The doses were referred to previous studies done by other authors [15] and us (in press). Under these low initial doses, peri-implant drug concentration could keep at a low level for a long period that locally released BPs could inhibit osteoclast activity without inhibiting osteoblast activity [4-6]. 100-μg/50-μg of locally administrated ALN/RIS in a rabbit equates approximately to 50-mg/25-mg of orally administrated ALN/RIS in an adult, which are lower than their weekly doses.

### Animals and surgery

Animals were anaesthetised with an intramuscular injection of ketamine (35 mg/kg) and xylazine (5 mg/kg). After an incision was made to the left knee joint, a 2.5-mm diameter bone tunnel was drilled through the tibia plateau into the medullary cavity. Then 2 × 10^8^ UHMWPE particles (suspended in 200-μL of saline) were injected around implants to induce a high bone turnover environment. Buprenorphine (0.04 mg/kg) was administered subcutaneously every 6–12 h for two days after surgery for postoperative pain control. Unrestricted activity was allowed after surgery. Antibiotics were administered 1 day preoperatively and 3 days postoperatively. On days 14 and 4 before being killed, the animals received tetracycline (25 mg/kg) injections to label the newly formed bone.

**Figure 1:**
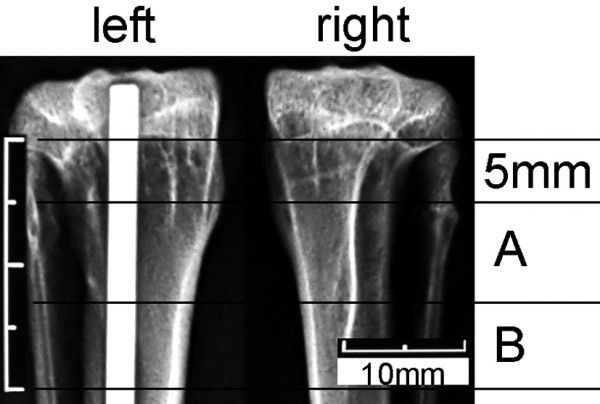
**A radiograph of bilateral tibias (group I) retrieved at week 12 postoperatively.** Levels (**A** and **B**) of the consecutive cross-section specimens.

After sacrifice, bilateral tibias and lumbar vertebrae (L2 and L3) were retrieved and fixed in 70% ethanol for 7 days. After fixation of the tibias, serial cross-sections (Figure 1) were cut using a low speed saw (IsoMet, Buehler LTD, Lake Bluff, IL). An 8-mm-thick cross-section (section A) was cut 5-mm below the proximal epiphyseal growth plate for dynamic histomorphometric evaluation, and a 7-mm-thick cross-section (section B) was cut for micro-CT scanning and push-out test. The protocol was approved by our Institutional Animal Care and Use Committee.

### Histomorphometry

Dynamic bone histomorphometry. Section A of bilateral tibias (Figure 1) and the L2 vertebrae were embedded in methylmethacrylate and two serial 15-μm thick slices of each section were made using a hard tissue microtome (Leica SP1600, Leica, Nussloch, Germany). They were processed for toluidine blue staining and fluorescence microscopy. The parameters were expressed in accordance with the American Society of Bone and Mineral Research (ASBMR) nomenclature, viz., bone formation rate (BFR/BV) and mineral apposition rate (MAR).

Static bone histomorphometry. The BIC was defined as the ratio of the implant surface covered with bone, and it was measured using toluidine blue-stained slices. Section B of bilateral tibias (Figure 1) and the L3 vertebrae were scanned using an eXplore Locus SP micro-CT system (GE Healthcare, Healthcare, Milwaukee, WI). The scanning protocol was set at 80 kV and 80 μA with an exposure time of 3000 ms and the resolution of 21 × 21 × 21 μm. The scanning protocol was referred to previous studies [19,20], the manual instruction of the micro-CT system, and our previous study (in press). This protocol can minimise the influence of scanning artifacts induced by metal implants. The image data were analysed using GEHC MicroView software to measure BMD and static bone histomorphometric parameters, viz., bone volume fraction (BV/TV), trabecular thickness (Tb.Th), trabecular separation (Tb.Sp), and structure model index (SMI). Micro-CT was used to measure the static histomorphometric parameters because it is based on 3D imaging, which can reduce measurement errors.

### Push-out test

Following micro-CT scanning, the biomechanical properties of section B of the left tibias were analysed using an Autograph AGS-J (SHIMADZU, Japan) universal test machine. The whole specimen was placed vertically on a metal jig with a central opening, and the implant was pushed out of the bone. A preload of 2 N defined the start of the test, and the pushing speed was 0.5 mm/min. During the pushing period, load–displacement curves were generated. The detailed protocol was implemented according to the studies by Dhert *et al.*[29] and Jakobsen *et al.*[16]. The maximum force (MF) was defined as the peak value of the load–displacement curve, and apparent shear stiffness (ASS) and total energy absorption (TEA) were defined as the slope of the linear section of the curve and the area under the curve until failure, respectively.

### Bone turnover markers

Serum levels of B-ALP (ADL, USA), TRACP-5b (SBA Sciences, Finland), OPG and RANKL (R&D systems, USA) were measured with enzyme-linked immunosorbent assay (ELISA) kits according to the manufacturers' instructions at weeks 12 and 24 post-operatively. The intra- and inter-assay CVs for each assay were lower than 7.6% and 5.3% (B-ALP), 8.0% and 6.5% (TRACP-5b), and 6.7% and 5.5% (OPG and RANKL), respectively. All the samples were tested in duplicate.

### Statistical analysis

One-way ANOVA and Student-Newman-Keuls (*SNK-q*) tests were performed for multiple comparisons among all groups. A *p* value less than 0.05 was considered significant, and data were expressed as means ± standard deviations (SDs).

## Results

No complications occurred during the experimental period.

### Bone-implant contact

**Figure 2:**
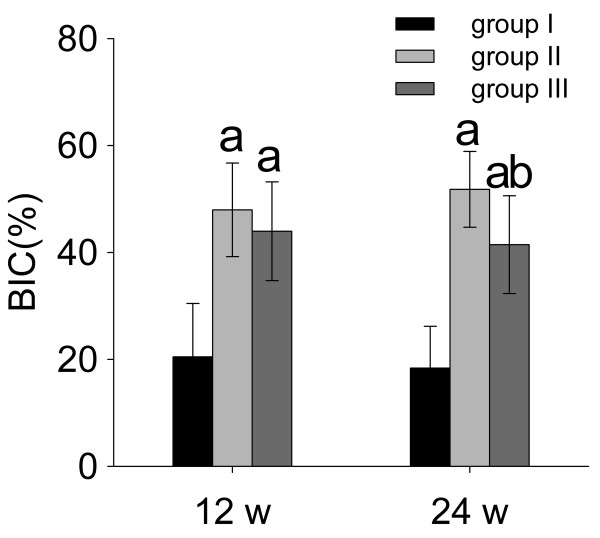
**Bone-implant contact curves. a**: significant vs. group I, **b**: significant vs. group II. Data were expressed as means ± SDs. BIC: bone-implant contact.

As shown in Figure 2, both ALN- and RIS-HA composite coatings could increase BIC ratios significantly at each time point (*p* < 0.05). At week 24, ALN (BIC = 51.82 ± 7.09%) showed stronger osteo-integrating effect compared to RIS (BIC = 41.46 ± 9.15%, *p* < 0.05).

### Bone histomorphometry

**Figure 3:**
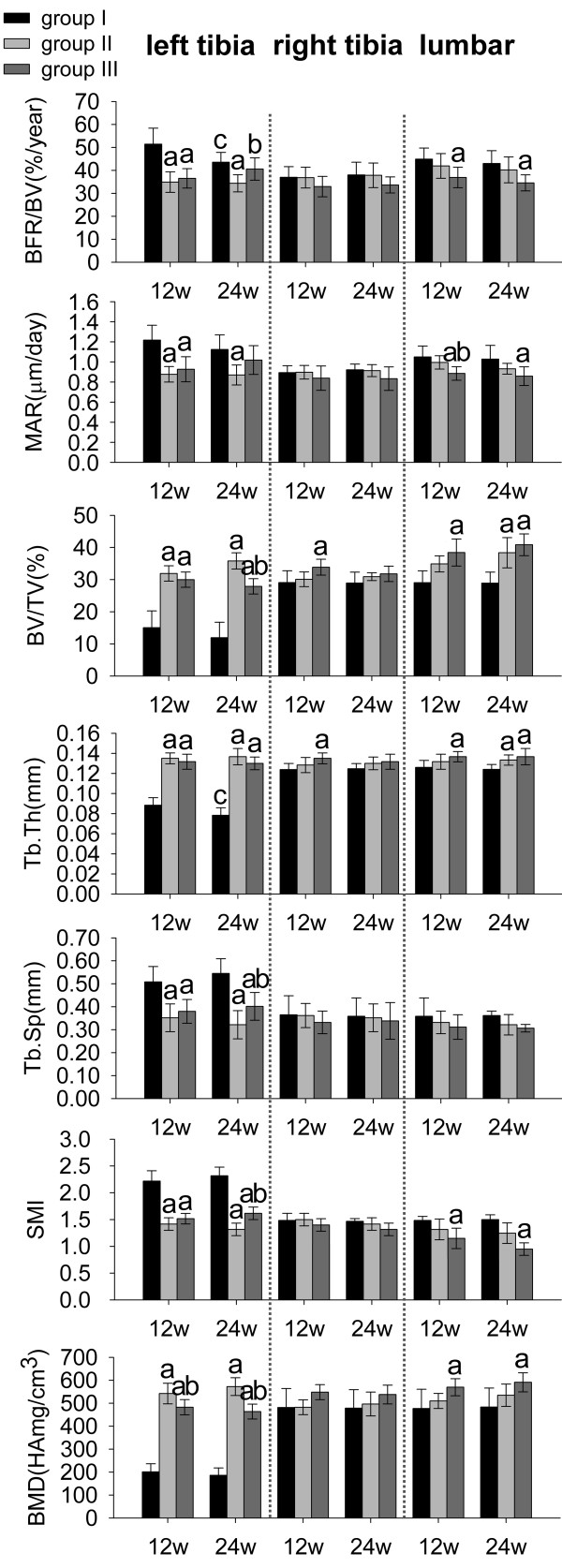
**Histomorphometric parameters and bone mineral density. a**: significant vs. group I, **b**: significant vs. group II, **c**: within-group significant vs. week 12. Data were expressed as means ± SDs. BFR/BV: bone formation rate, MAR: mineral apposition rate, BV/TV: bone volume fraction, Tb.Th: trabecular thickness, Tb.Sp: trabecular separation, SMI: structure model index.

Dynamic bone histomorphometry. As shown in Figure 3, both ALN and RIS reduced peri-implant (left tibia) BFR/BV (−32% and −29%) and MAR (−28% and −24%) significantly at week 12. However, only ALN reduced peri-implant BFR/BV (−21%) and MAR (−22%) significantly at week 24 (*p* < 0.05). Although RIS treatment resulted in the lowest BFR/BV and MAR in right tibias, it showed no significant differences between three groups. Only RIS reduced BFR/BV (−18% at week 12 and −20% at week 24) and MAR (−15% at week 12 and −17% at week 24) significantly in lumbar vertebrae (*p* < 0.05).

Static bone histomorphometry. As shown in Figure 3, both ALN and RIS treatment resulted in significantly higher peri-implant BV/TV (+112% and +99% at week 12, +199% and +133% at week 24, respectively) and Tb.Th (+53% and +50% at week 12, +76% and +67% at week 24, respectively) and lower Tb.Sp (−31% and −25% at week 12, -42% and −27% at week 24, respectively) and SMI (−36% and −32% at week 12, -43% and −30% at week 24, respectively) compared to the control group at each time point (*p* < 0.05), and ALN was more effective than RIS. However, ALN did not have significant effect in right tibias. Compared to the other groups, RIS treatment resulted in the highest BV/TV and Tb.Th and the lowest Tb.Sp and SMI in right tibias and lumbar vertebrae at each time point. However, the effect of RIS became insignificant in right tibias at week 24.

**Figure 4:**
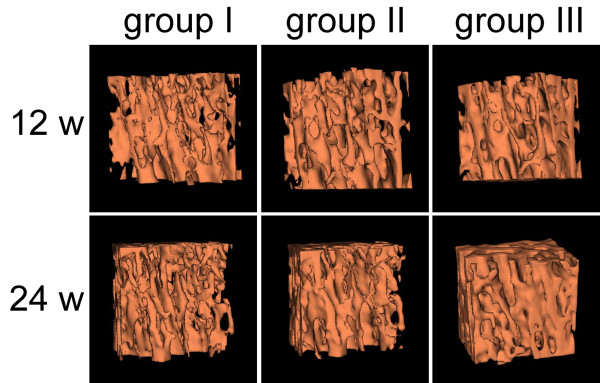
**Bone architecture of L3 vertebrae reconstructed by micro-CT at weeks 12 and 24 postoperatively.** RIS could induce higher lumbar vertebral bone mass augmentation compared to ALN.

Micro-CT scanning (Figure 4) showed obvious lumbar vertebral bone mass augmentation in RIS-treated animals, and these trabecular bones tended to be plate-like in structure. However, ALN did not have the equivalent effect on lumbar vertebrae.

### Bone mineral density

As shown in Figure 3, both ALN and RIS treatment resulted in significant peri-implant BMD augmentation, and ALN was more effective than RIS (+12% at week 12 and +24% at week 24, *p* < 0.05). Although RIS treatment resulted in the highest BMD augmentation in right tibias, the effect of neither ALN nor RIS was significant. In lumbar vertebrae, only RIS treatment resulted in significant higher BMD augmentation than the control group (*p* < 0.05).

### Implant stability

**Figure 5:**
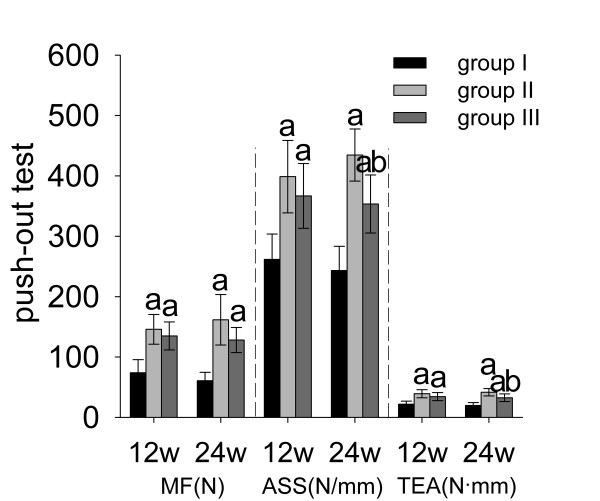
**Push-out test. a**: significant vs. group I, **b**: significant vs. group II. Data were expressed as means ± SDs. MF: maximum force, ASS: apparent shear stiffness, TEA: total energy absorption.

As shown in Figure 5, both ALN and RIS improved implant stability significantly at each time point (*p* < 0.05). It was more obvious in ALN-treated animals at week 24 that ALN treatment resulted in higher ASS (+19%) and TEA (+13%) at week 24 compared to RIS treatment (*p* < 0.05).

### Bone turnover markers

**Figure 6:**
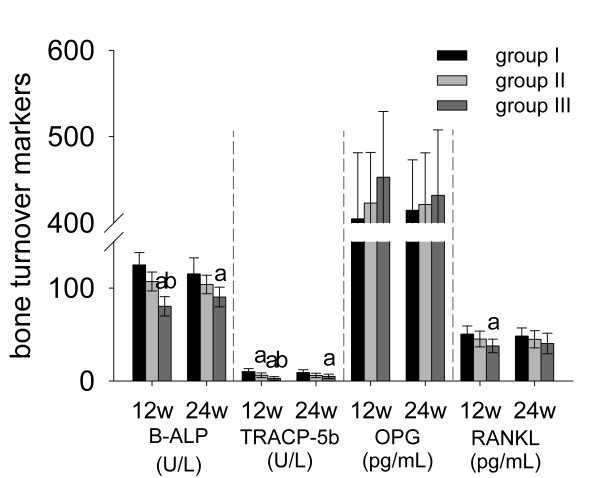
**The level of bone turnover makers measured by ELISA. a**: significant vs. group I, **b**: significant vs. group II. Data were expressed as means ± SDs.

As shown in Figure 6, serum levels of both B-ALP (−36% at week 12 and −22% at week 24) and TRACP-5b (−77% at week 12 and −45% at week 24) reduced significantly in group III at each time point and it was more obvious at week 12 than week 24. However, ALN could only reduce TRACP-5b (−38%) significantly at week 12 (*p* < 0.05). Serum levels of OPG and RANKL showed no significant differences between three groups except the significantly reduced serum level of RANKL (−25%) by RIS treatment at week 12.

## Discussion

Locally released BP from either ALN- or RIS-HA composite coating could induce peri-implant bone mass augmentation, BMD increment, and the improvement of bone architecture, which significantly improved the bone-implant integration (BIC increment), at each time point (Figures 2, 3 (left tibia) and 4).

Early bone-implant integration that can provide a sealed interface is important to implant stability because it can inhibit the migration of debris and cytokines [13,30]. In addition, implant stability is also influenced by peri-implant bone architecture. Architecturally compromised trabecular bone will transmit a lesser load to the cortical bone. Thus, stress will concentrate at the bone-implant interface, which ultimately results in implant loosening. Rod-like trabecular bone (SMI = 3) had poorer biomechanical properties than plate-like trabecular bone (SMI = 0). After the particle stimulation, SMI of the peri-implant bone was higher than 2.0 (Figure 3, SMI, left tibia, group I). However, locally released BP from BP-HA composite coatings significantly reduced SMI to almost 1.5 (Figure 3, SMI, left tibia, groups II and III) and led to higher implant stability, which was confirmed by the results of push-out test (Figure 5).

It was obvious that ALN was more potent than RIS on peri-implant bone due to their different mineral-binding affinities [8], especially at week 24. ALN has high mineral-binding affinity and intermediate inhibitory potency on FPPS. It can keep a stable concentration in peri-implant bone and will hardly be delivered far away. Therefore, previous studies [1,2,4,12-20,31,32] on enhancing bone-implant integration almost focused on such high-affinity BPs. Oppositely, RIS has low mineral-binding affinity and high inhibitory effect on FPPS [7]. Such low-affinity BPs will be delivered to the entire osteocyte network through the canalicular compartment by extracellular fluid [3] that effective peri-implant drug concentration may not be sustained for a long lime. Therefore it is used for treating non-vertebral fractures but not for enhancing bone-implant integration [3,11].

However, as we know, revision patients are averagely older than the primary patients that they have a higher incidence rate of systemic osteopenia or osteoporosis. Therefore, in addition to enhancing implant stability, it is better for the composite coatings to have systemic effects of bone mass augmentation and BMD increment which may benefit the recovery of revision patients. However, the HA coating composed with high-affinity BPs may not fit the needs as Jakobsen *et al.*[16] confirmed that locally released ALN did not have systemic effects. Thus, we should re-consider the possibility of using low-affinity BPs as coating materials.

The histomorphometric and BMD results of the contralateral tibias and lumbar vertebrae (Figure 3) indicated that RIS-HA composite coating could induce bone mass augmentation and BMD increment significantly in non-peri-implant region, especially lumbar vertebrae. Oppositely, ALN had very limited systemic effects. This difference suggests that low-affinity BPs are more systemically effective than high-affinity BPs. Additionally, the variations of serum cytokine levels can reflect their systemic effects more directly and precisely compared to histomorphometric parameters. The significant lower serum concentrations of B-ALP, TRACP-5b and RANKL in group III than in groups I and II at week 12 also supported our histomorphometric results (Figure 6). The effects of RIS were more pronounced in lumbar vertebrae than contralateral tibias (Figure 3), because BPs tend to bind with the bone which has the highest turnover rate [25]. Kimmel *et al.*[33] confirmed that lumbar vertebrae and proximal humerus have the highest turnover rates.

Local BP administration (BP-HA composite coating) is the best way for enhancing implant stability and preventing aseptic loosening. Because of the binding selectivity of BPs, they will almost concentrate in the bone with the highest turnover rate, such as the lumbar vertebrae, but not in the peri-implant bone if they are administrated systemically. The drug potency is then diminished. Especially in the osteoporosis patients who are usually in the state of systemic high bone turnover, BP is more likely to be consumed by non-peri-implant bone. Additionally, local administration has many advantages such as lower initial dosage, higher peri-implant drug concentration and fewer side effects compared to systemic administration.

For inhibiting peri-implant bone resorption, low-affinity BPs have some limitations that peri-implant drug concentration may not sustain for a long time and their effects on peri-implant bone will become weaker than high-affinity BPs. In our study, it is obvious that BIC, BV/TV, Tb.Sp, SMI, ASS, and TEA in group III became significantly worse than in group II at week 24, and BFR/BV and MAR showed no significant differences compared to the control group (Figures 2,3 (left tibia) and 5). Therefore, low-affinity BP alone is not suitable for revision patients who suffer from osteoporosis simultaneously. Composing HA with both high- and low-affinity BPs may be feasible. Recently, Abtahi *et al.*[21] composed both high- (pamidronate) and low-affinity (ibandronate) BPs to the fibrinogen matrix as the coating material, and each oral implant was then screwed into the upper jaw in five patients. The results indicated that this composite coating could successfully improve implant stability. However, they did not explain why they composed two kinds of BPs, and they did not compare it with single BP composed coatings.

The mechanism by which BPs released from either pure HA *in vitro* or bone *in vivo* are crucial to further studies on composite HA coatings. In addition to different mineral-binding affinities, the release duration of BPs also depends on the bone remodelling activity that BPs will be consumed more rapidly in the sites with high turnover rates. Thus, for further evaluating peri-implant concentration and the release duration of BP-HA composite coatings, detecting drug concentration in plasma or bone is meaningful. Tanzer *et al.*[1] used a spectrophotometric method [1,34] to investigate the *in vitro* release characteristic of ZOL from pure HA but it cannot precisely reflect the release duration *in vivo*, because in addition to chemical desorption, osteoclastic resorption also plays an important role. Legay *et al.*[35] developed a highly sensitive radioimmunoassay (RIA) method to detect ZOL concentration, and the limit of quantification was 0.4 ng/mL in plasma and 5 ng/mL in urine, respectively. Yun e*t al.*[36] developed a high-performance liquid chromatography (HPLC) method to detect ALN concentration, and the limit of quantification was 1 ng/mL in plasma. These methods are not sensitive enough for our study because the doses of ALN and RIS were 100-μg and 50-μg, respectively, per implant, that the drug concentration was too low to detect, especially in plasma. Recently, Stadelmann *et al.*[22] developed a novel mathematic-based modelling method for evaluating peri-implant BP concentration and bone density. Using this method, they found the optimal coating dose of ZOL per implant. All these efforts mentioned above will help us further realise the characteristic of BP-HA composite coatings.

The results of this study are instructive and meaningful to further clinical studies that we could choose different BP-HA composite coatings according to the patient’s condition. In brief: 1) In primary TJA patients without systemic high bone turnover-related diseases (osteopenia or osteoporosis), we can prefer ordinary non-BP-composed prostheses; 2) In primary TJA patients with systemic high bone turnover-related diseases, we can prefer low-affinity BP-composed prostheses. Their significant systemic effects may cooperate with the BP which is systemically administrated. The systemic bone mass augmentation and BMD increment can result in earlier movement and shorter recovery time for patients; 3) In revision patients without systemic high bone turnover-related diseases, we can prefer high-affinity BP-composed prostheses which can significantly reduce peri-implant high bone turnover rate and improve implant stability due to the stable peri-implant drug concentration and long release duration; 4) In revision patients with systemic high bone turnover-related diseases, the HA coating composed with both high- and low-affinity BPs may be preferable, in addition to improving implant stability, it can result in systemic bone mass augmentation and BMD increment.

Nevertheless, our study has some limitations. Firstly, the homogenous distribution of the BP solution on the implant surface could be guaranteed due to the implant size and the BP solution volume in our study. However, in further studies, if the implant is bigger or the solution amount is less, the homogenous distribution may not be guaranteed. Therefore, other loading methods like spraying may be possible if the drug amount can be controlled at the same time. Secondly, we compared local and systemic inhibitory effects on bone resorption only between high- and low-affinity BPs. In further studies, an additional group that the HA coating is composed with both high- and low-affinity BPs should be added and an appropriate ratio of high-/low-affinity BPs should be investigated. Finally, the animal model used in this study was to induce peri-implant (local) high bone turnover. In further studies, these composite coatings should be studied in osteoporosis models which have systemic high bone turnover rate.

## Conclusions

In this long-term study, we developed two kinds of BP-HA composite coatings using either high- or low-affinity BPs in rabbits. The results indicated that ALN-HA composite coating was more effective on peri-implant bone, while RIS-HA composite coating was more effective on non-peri-implant bone, especially lumbar vertebrae. It is instructive and meaningful to further clinical studies that we could choose different BP-HA composite coatings according to the patient’s condition.

## Competing interests

The authors declare that they have no competing interests.

## Authors’ contributions

SN and XC participated in the experimental design, surgery process, histomorphometric examinations and push-out tests. YZ was responsible for animal care and performed the ELISA assays and statistical analysis. This study and its design were conceived by QZ, JZ, and PZ, who coordinated the project, participated in the experimental design. All authors contributed to the drafting of the manuscript, and have read and approved the final manuscript.

## Pre-publication history

The pre-publication history for this paper can be accessed here:

http://www.biomedcentral.com/1471-2474/13/97/prepub

## Supplementary Material

Additional file 1 **BP-HA composite coating.** The top figure shows the HA-coated implant which has not been loaded, and we can see the HA coating is dry. After loading with 100-μL of BP solution, as shown in the bottom figure, the implant surface is entirely covered with the solution. We rotated the implants (90 degrees) every 5 minutes until there was no dripping (arrow) on the implant surface. Click here for file
