# Supplementary figures and images for: Peri-implant and systemic effects of high-/low-affinity bisphosphonate-hydroxyapatite composite coatings in a rabbit model with peri-implant high bone turnover
Source: BMC Musculoskelet Disord. 2012 Jun 11;13:97. doi: 10.1186/1471-2474-13-97 (PMC3414815; doi:10.1186/1471-2474-13-97)

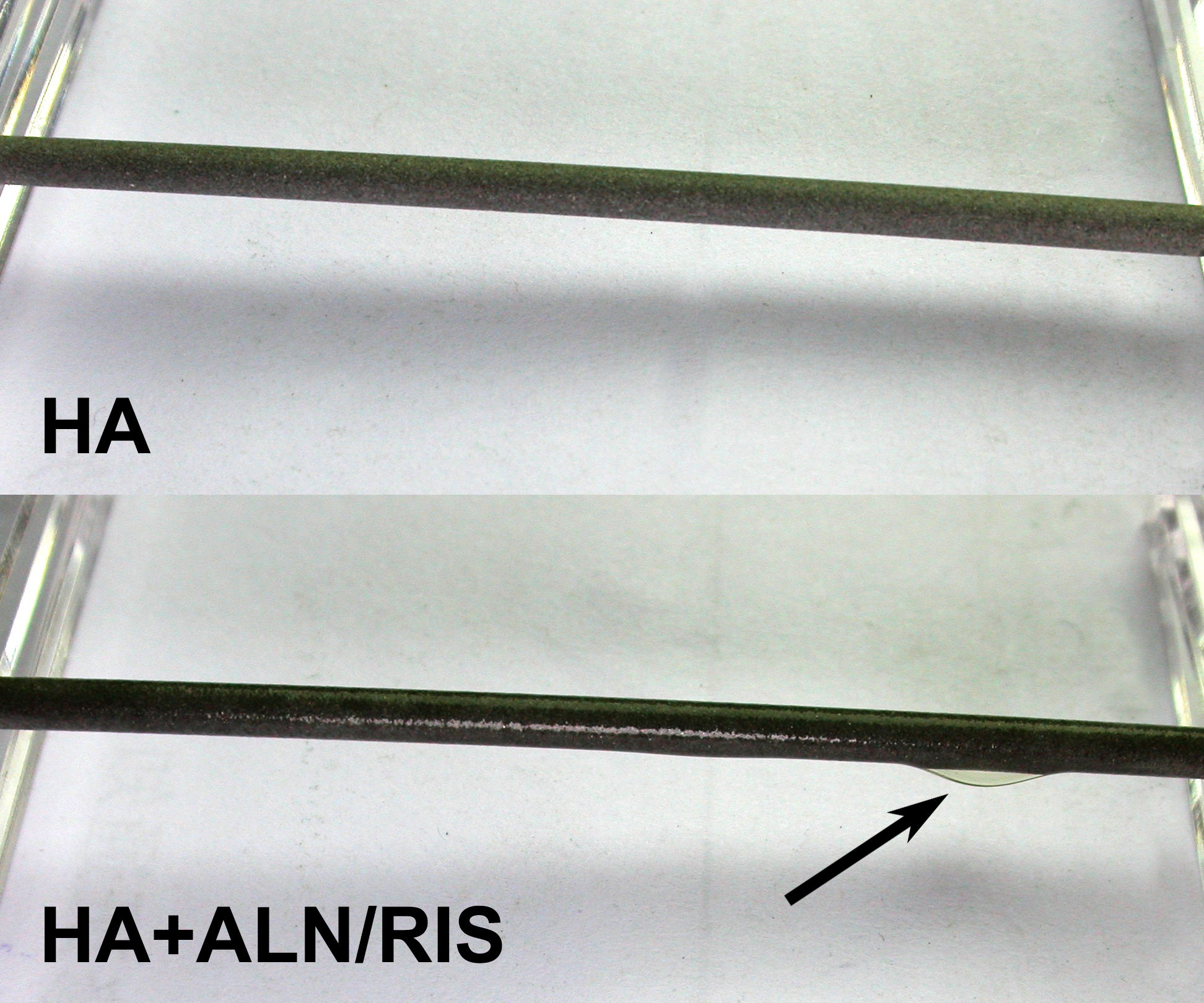

Supplement: Additional file 1 — BP-HA composite coating. The top figure shows the HA-coated implant which has not been loaded, and we can see the HA coating is dry. After loading with 100-μL of BP solution, as shown in the bottom figure, the implant surface is entirely covered with the solution. We rotated the implants (90 degrees) every 5 minutes until there was no dripping (arrow) on the implant surface. [file 1471-2474-13-97-S1.jpeg]
